# Dietary Patterns in the Argentinian Population and Their Association With Sociodemographic Characteristics: Results From the ELANS Study (2014–2015)

**DOI:** 10.3389/fnut.2022.778390

**Published:** 2022-03-03

**Authors:** Ignacio Mendez, Maria Victoria Fasano, Viviana Guajardo, Luciana Zonis, Irina Kovalskys

**Affiliations:** ^1^Instituto de Desarrollo e Investigaciones Pediátricas (IDIP), Hospital de Niños Sor María Ludovica, Buenos Aires, Argentina; ^2^Centro de Matemática de La Plata, Facultad de Ciencias Exactas, Universidad Nacional de La Plata/Comisión de Investigaciones Científicas, Buenos Aires, Argentina; ^3^Instituto para la Cooperación Científica en Ambiente y Salud (ICCAS), Buenos Aires, Argentina; ^4^Escuela de Nutrición, Facultad de Ciencias Médicas, Pontificia Universidad Católica Argentina, Buenos Aires, Argentina

**Keywords:** Argentina, Prudent diet, western diet, dietary patterns, sociodemographic

## Abstract

**Aim:**

To describe dietary patterns in the Argentinian population and their association with sociodemographic characteristics.

**Methods:**

Cross-sectional analysis of Argentina's food consumption and sociodemographic data of 1,266 men and women between 15 and 65 years from the Latin American Study of Nutrition and Health carried out between March 2014 and December 2015. Dietary patterns were identified by using factor analysis and median factor scores were calculated for gender, age, region, body mass index (BMI), socioeconomic, education, and physical activity categories.

**Results:**

Five dietary patterns were identified. Western, Local Western, and Rural were generally characterized by high consumptions of animal fats, sugar-sweetened beverages, meats or processed meats, pizza and empanadas, sweets, pastries, and low consumption of fruits and vegetables. Traditional pattern was mainly characterized by consumption of oils, starchy vegetables, and red meat and Sweet Prudent by milk and yogurt, vegetables, fruit, artificially sweetened beverages, sweets, and cookies. Higher adherence to the Sweet Prudent pattern was observed in women, in people who meet physical activity guidelines and higher socioeconomic and educational levels. Higher adherence to traditional pattern was only observed for men. Higher adherence to the rest of the patterns was observed mostly by men, young, leaner, lower socioeconomic, and educational levels, not meeting physical activity guidelines, from the metropolitan area of Buenos Aires or northern regions.

**Conclusion:**

Food consumption in Argentina is expressed in a diversity of dietary patterns. Men, younger, and sedentary individuals, with lower socioeconomic and educational level, from the metropolitan area of Buenos Aires and northern regions, seem to have higher adherence to least healthy dietary patterns.

## Introduction

Diet is among the most important modifiable risk factors for morbidity and mortality (1). Understanding population food consumption is a key to promote changes in dietary habits as well as identifying the risk groups.

Historically, study has focused on individual food or nutrients as well as other reductionist metrics such as calories or total fats (2). Nevertheless, food consumption occurs in different complex combinations of diverse foods and beverages across time to compose the overall diet. For this reason, nutrition epidemiology has shifted from individual food and nutrients to dietary pattern analysis (3, 4).

Two major approaches have been described for this purpose (5, 6). A priori-based dietary pattern analysis uses scores or indexes to measure subject adherence to a predefined dietary pattern. A wide variety of indexes have been developed that commonly assess adherence to the Mediterranean diet, diet diversity, or compliance with dietary guidelines (7, 8).

On the other hand, a posteriori approach uses different methods such as cluster analysis, factor analysis (FA), and principal component analysis to extract dietary patterns from food consumption data, thus proving information on existing dietary patterns in a given population (5, 6). Particularly, FA allows extracting latent variables or factors that represent the eating habits of the population, based on the correlation of food consumption variables (9).

There are also hybrid methods such as reduced rank regression that allow extracting dietary patterns related to predictor variables of interest, on the basis of previously known diet-disease relationships (5, 10).

Current knowledge of Argentinian dietary habits is limited to mean quantities of individual foods or the food groups provided by balance sheets (11), household food consumption data (12), or cross-sectional nutrition surveys (13, 14). Few studies have explored dietary patterns. For example, one study explored dietary patterns in the Andean population from the province of Jujuy. It found two major dietary patterns, “not-Autochthon/Western-like”, positively associated with consumption of beef, chicken, bread, and sugar-sweetened beverages (SSBs) and negatively associated with consumption of more local foods such as tubers, tortillas, lamb, and llama. The “Autochthon, Andean-like” pattern was associated with the consumption of herbal infusions, cereals, legumes, tubers, and vegetables (15). Another study described dietary patterns from 489 participants on a case–control cancer study from the province of Cordoba. Three major dietary patterns were described as “Southern Cone” associated with consumption of red meat, wine, and starchy vegetables, “sugar beverages” associated with consumption of soda and juice, and “Prudent” associated with consumption of dairy and fruits and vegetables (16). Other studies that used a posteriori dietary patterns methods were mostly limited to the province of Cordoba (17–22). National representative data describing dietary patterns is still lacking.

The Latin American Study of Nutrition and Health/Estudio Latinoamericano de Nutrición y Salud (ELANS) is a multicenter cross-sectional nutrition and health surveillance study of a nationally representative sample of urban populations from eight Latin American countries including Argentina (23). Therefore, data from the ELANS provide an opportunity to better understand eating habits through dietary pattern analysis.

This study aimed to describe dietary patterns in the Argentinian population and their association with sociodemographic characteristics.

## Materials and Methods

The ELANS is an epidemiological study, whose design focused on evaluating dietary intake and physical activity (PA) in a representative sample of the urban population of eight Latin American countries (Argentina, Brazil, Chile, Colombia, Costa Rica, Ecuador, Peru, and Venezuela). For this study, we worked with the data corresponding to the Argentine Study of Nutrition and Health (EANS). The detail of the study design is published in a study by Fisberg et al. (23).

### Population

The final sample for Argentina was made up of 1,266 individuals and was stratified by gender, age, geographic region, and SEL. The sample consisted of men and women, between 15 and 65 years old, living in urban areas of the most densely populated regions selected through complex multistage random sampling. Only urban areas were included in order to maintain a homogeneous population for the study and based on the fact that almost all the countries have at least 80–90% of their population living in urban areas. Individual quotas were defined for each of these variables, which allow for the identification of the total number of interviews required to adequately represent the sociodemographic distribution in this study. The survey was designed, so that no more than one subject was selected from a household.

The sample size was calculated with a confidence level of 95% and a maximum error of 2.83%. A survey design effect of 1.75 was estimated based on the guidance of the US National Center for Health Statistics and calculations were made of the minimum required sample sizes by strata (i.e., socioeconomic status, age, and gender) for each country (23).

All the participants gave their informed consent/assent to participate in this study. Those who had any chronic or acute disease that affected their eating behavior were excluded as well as pregnant and lactating women (with children under 6 months of age). For the classification by SEL, a questionnaire of wide local use was applied, proposed by the Institutional Liaison Commission AAM-SAIMO-CEIM, which classifies SEL into seven categories that were regrouped into three (high, medium, and low) (24). The final sample is representative of each region and was weighted based on the information available from the 2010 Population Census and the 2015 Permanent Household Survey, adjusted to the population projections for the year 2015 (25, 26).

### Data Collection

The ELANS data collection process was carried out at home in two visits. In the first visit, the participants were given a questionnaire on sociodemographic data (age, SEL, educational level, etc.) and the first 24-h recall (R24). The second visit was carried out 3 to 8 days after the initial contact and in this visit, the second R24 was carried out. The R24 was performed with a standardized intake collection technique (multistep technique) performed by an interviewer trained for this purpose to improve the precision of the information obtained. The multistep technique is a tool validated by the US Department of Agriculture (27). A pilot test was conducted with this tool for this study. It is mentioned in the several ELANS publications. Support materials were used (e.g., visual guide of portions and measurements specifically designed for local foods and utensils) (28), which allowed determining the portions more precisely, a team of trained nutritionists recorded food and beverages in household measurements, and then converted them into grams and milliliters. Inconsistencies or missing data were verified over the phone by an assistant nutritionist or the research team.

### Food Intake Analysis

The information obtained was loaded into the Nutrition Data System for Research (NDS) software (NDS software, version 2013, NDS-R, Minnesota University, Minnesota, USA), through which the composition in energy, macronutrients, and micronutrients of the total food and drinks is obtained. The preparations or recipes that are not contained in the program's database were loaded by their ingredients. Specifically, for Argentine foods, 638 foods and 195 local recipes were standardized.

Complete information on the study's food intake standardization and evaluation methodology can be found in a study by Kovalskys et al. (29). For this analysis, the selected food and beverage groups were classified into 22 categories: fruits and natural juices, vegetables (including all the raw, cooked, frozen, or canned, excluding starchy vegetables), starchy vegetables, cereals (rice and pasta), pizza and empanadas (they have been analyzed independently of cereals, as they are widely consumed by the Argentine population), bread, baked goods, pastries, cookies, and crackers. Cookies and pastry foods are the separate groups, since they are also Argentine cultural foods; red meats, processed meats (including hamburgers, sausages, cold cuts, etc.), and poultry. Dairy products (milk and yogurt), water, SSBs, infusions with added sugar, artificially sweetened beverages (ASBs), alcoholic beverages, sweets and treats, vegetable oils, and animal fats.

### Anthropometry

The anthropometric variables considered were: weight and height, which were taken from the individual at home, during the first visit. Bodyweight (to the nearest 0.1 kg) was measured with the Seca® Portable Scale (Hamburg, Germany, UK) up to 200 kg, after removing all the heavy clothing, pocket items, and shoes. Height was measured with the Seca 213® Portable Stadiometer (Hamburg, Germany, UK) at the end of a deep inhalation with the participant's head in the Frankfort plane, whose measurement range was 0–205 cm (30). The body mass index (BMI) was calculated in kg/m^2^ (31). Subjects were classified according to their BMI as normal/underweight (BMI <25 kg/m^2^), overweight (BMI 25–29.9 kg/m^2^), and obese/morbidly obese (BMI > 30 kg/m^2^). Two measurements of the anthropometric variables were made and the average was used for the analysis. The detail of this information is published in a study by Fisberg et al. (23).

### Physical Activity

To collect the information, the International Physical Activity Questionnaire (IPAQ) was used in its long version in Spanish, which was adapted for Argentina, with terms and examples adjusted to our daily reality (32). The IPAQ is designed to assess levels of habitual PA in people aged 15 to 69 years (33, 34). For this study, adults who complied with 150 min of PA/day and adolescents who complied with 60 min of PA/day were considered as active and those who did not comply with the said recommendation were considered as inactive (35).

### Statistical Analysis

Statistical analysis was performed with R package version 4.0.3 (R Foundation for Statistical Computing, Vienna, Austria). Descriptive statistics were calculated for each sociodemographic variable by using frequency and percentages. Dietary patterns were obtained by robust exploratory FA for the 22 food groups. As the variables had different units, we first standardized the data by using the MM robust location and covariance estimates (36) from the R package rrcov. To evaluate multicollinearity and sampling adequacy, the Bartlett's test of sphericity and the Kaiser–Meyer–Olkin (KMO) measure were applied to the robust correlation matrix. The KMO value above 0.50 was considered as acceptable.

Robust exploratory FA was then performed to identify the dietary patterns (R package robustfa). Principal component analysis was used for extraction of factors and Varimax orthogonal rotation to simplify their structure and to facilitate their interpretability. The number of factors to retain was determined according to the following criteria: detection of an inflection point in the scree plot, factor eigenvalues >1.2, and the interpretability of the factors.

A rotated factor loading matrix was constructed to assess the strength and direction of the associations between the factors and food groups. The food groups with absolute factor loadings >0.20 were used to name the patterns. The factor score for each pattern was constructed by summing up observed intakes of the component food items weighted by the factor loading. Higher factor scores indicate a higher consumption of the food group, while lower factor scores indicate a lower consumption of the food group.

Median factor scores were then calculated for the sociodemographic variables: gender, region, BMI, age, SEL, education, and PA and were tested for significant differences across groups by using the Mann–Whitney *U* test for two levels or the Kruskal–Wallis test when more than two levels. Pairwise comparisons between the multiple groups were adjusted by the Benjamini and Hochberg (BH) method. For all the statistical tests, *p* < 0.05 was considered as statistically significant.

### Ethical Aspects

The ELANS has been approved by the Western Institutional Review Board (# 20140605) and registered with ClinicalTrials (# NCT02226627). In Argentina, the approval of the ethics committee of the Argentine Medical Association has been added for the local approval of the international study. Study participants completed an informed consent before participation and a consent plus assent for cases 15.0 to 17.9 years.

## Results

### Sociodemographic Characteristics

A description of the sociodemographic characteristics of the study sample is given in Table 1. The average age of the population under study was 36.8 (SD 13.9) years, with a higher proportion of women (54.7%) than men (45.3%). The majority of the participants had received a basic education and predominantly had a low or medium SEL. A higher proportion of the sample belonged to the Metropolitan Area of Buenos Aires (AMBA) region (37%), followed by the Pampa region (29.5%), whereas a small proportion belonged to the Patagonia region (3.5%).

**Table 1:** Sociodemographic characteristics of Argentinian sample of the Estudio Latinoamericano de Nutrición y Salud (ELANS) study.

**Variable**	**Category**	**Frequency (%)**
Total sample		1,266
Gender	Men	573 (45.3%)
	Women	693 (54.7%)
Geographic region^*^	Metropolitan Area of Buenos Aires (AMBA)	468 (37.0%)
	Pampa	374 (29.5%)
	Cuyo	103 (8.1%)
	Northwest	138 (10.9%)
	Northeast	139 (11.0%)
	Patagonia	44 (3.5%)
BMI categories	<25 kg/m^2^	530 (41.9%)
	25–29.9 kg/m^2^	399 (31.5%)
	≥ 30 kg/m^2^	337 (26.6%)
Age group	15–19	152 (12.1%)
	20–34	446 (35.2%)
	35–49	379 (29.9%)
	50–65	289 (22.8%)
Socioeconomic level	High	65 (5.1%)
	Medium	585 (46.2%)
	Low	616 (48.7%)
Education level	High (university degree)	54 (4.3%)
	Medium (high school)	257 (20.3%)
	Low (basic)	955 (75.4)
Meeting physical activity guidelines (*n* = 1,224)	No	588 (46.4%)
	Yes	636 (50.2%)

* *Metropolitan Area of Buenos Aires: Capital Federal and urban areas of Buenos Aires. Pampa: provinces of Buenos Aires, Córdoba, La Pampa and Santa Fe. Cuyo: provinces of San Luis, San Juan and Mendoza. Northwest: provinces of Jujuy, Catamarca, Tucumán, La Rioja, Salta and Santiago del Estero. Northeast: provinces of Misiones, Formosa, Chaco, Entre Ríosand Corrientes. Patagonia: provinces de Río Negro, Neuquén, Chubut, Santa Cruz and Tierra del Fuego*.

Data on PA was only available on 1,224 subjects and 48% of them did not meet PA guidelines. More than half (58%) of the population under study were overweight (BMI 25–29.9 kg/m^2^) or obese (BMI ≥ 30 kg/m^2^).

### Dietary Patterns

Factor analysis identified five dietary patterns from the 22 food groups. These factors explained 19.2% of the total variance in food intake. The rotated factor loading matrix is shown in Table 2. A positive loading indicates a positive association with the dietary pattern, whereas a negative loading indicates an inverse association. High loadings indicate strong associations between the corresponding food groups and the derived patterns. The food groups with absolute factor loadings >0.20 were considered to name the factors. Factor one (named “Western pattern”) was positively associated with the following food groups: animal fats, pizza and empanadas, pastries, processed meats, SSB, and sweets and treats. Factor two (named “Rural pattern”) was positively associated with the consumption of bread, SSB, processed meats, cereals, alcoholic beverages, whereas it was negatively associated with the consumption of crackers, ASB, and fruits. Factor three (named “Traditional pattern”) was characterized by the consumption of oils, starchy vegetables, red meat, vegetables, poultry, alcoholic beverages, SSB, animal fats, and low consumption of cereals and crackers. Factor four (named “Sweet Prudent pattern”) was positively associated with the consumption of milk and yogurt, fruits, sweets and treats, ASB, cookies, and vegetables and negatively associated with the consumption of infusions with added sugar and baked goods. Finally, factor five (named “Local Western pattern”) was characterized by the consumption of SSB, cookies, poultry, starchy vegetables, pizza and empanadas, and low consumption of water, fruits, and vegetables. Specific foods contained in the food groups positively and negatively associated with each dietary patterns are shown in Table 3.

**Table 2:** Varimax rotation of the factor loading matrix for the major factors.

**Factor**	**Western**	**Rural**	**Traditional**	**Sweet Prudent**	**Local western**
Animal fats	0.85		0.11		
Pizza and empanadas	0.39				0.12
Pastries	0.33				
Processed meat	0.31	0.23			
Sugar-sweetened beverages	0.13	0.39	0.11		0.51
Sweets and treats	0.10			0.18	
Bread		0.69			
Crackers		−0.24	−0.10		
Cereals		0.18	−0.15		
Artificially sweetened beverages		−0.17		0.15	
Alcohol		0.12	0.12		
Fruits and fruit juice		−0.10		0.18	−0.27
Oils			0.62		
Starchy vegetables			0.48		0.13
Red meat			0.32		
Vegetables			0.14	0.11	−0.19
Poultry			0.13		0.18
Milk and yogurt				0.62	
Infusions with added sugar				−0.32	
Cookies				0.13	0.22
Baked goods				−0.12	
Water					−0.38
%Variance explained	5.3%	3.9%	3.9%	3%	3.1%

*Food groups with absolute factor loadings at least 0.20 were considered to name the factors. A positive loading indicates a positive association with the dietary pattern, whereas a negative loading indicates an inverse association. High loadings indicate strong associations between the corresponding observed variables and derived patterns.

**Table 3:** Foods positively and negatively associated with each dietary pattern.

**Dietary pattern**	**Foods positivly associated**	**Foods negatively associated**
Western pattern	Butter, cream or lard. Pastries, cakes and puddings. Pizza and empanadas^*^. Hamburger, sausage, bacon, salami, cold cuts. All kind of ready-to-drink beverage with added sugar (e.g.,: powder juice, nectar, sodas, energy drinks, teas, flavored water). Table sugar, candies, sweets and marmalades.	-
Rural pattern	Whole grain or refined grain. Rice (white or brown), refined grain, pasta (fresh and dry pastas: noodles, ravioli and gnocchi) whole grain, etc.	Commercial Crackers. All kind of ready-to-drink beverage with artificially sweeteners. All kind of fruits.
Traditional pattern	All kinds of vegetables oils (olive, corn, sunflower, etc.). Beef, pork meat, lamb. All kinds of vegetables including Potato, sweet potato, corn, cassava. Poultry. All kinds of alcoholic beverages (wine, beer, spirits, etc.). All kind of ready-to-drink beverage with added sugar (e.g.,: powder juice, nectar, sodas, energy drinks, teas, flavored water). Butter, cream or lard.	Rice (white or brown), refined grain, pasta (fresh and dry pastas: noodles, ravioli and gnocchi), whole grain, etc. Commercial Crackers
Sweet Prudent pattern	All milk and yogurt: (whole or skimmed). All kind of fruits. Table sugar, candies, sweets and marmalades. All kind of ready-to-drink beverage with artificially sweeteners. Sweet cookies: plain and filled. All kinds of vegetables (except starchy vegetables).	Tea, coffee and mate with added sugar. Buns, salty biscuits and other fatty breads.
Local Western pattern	All kind of ready-to-drink beverage with added sugar (e.g.: powder juice, nectar, sodas, energy drinks, teas, flavored water). Sweet cookies: plain and filled. Poultry. Potato, sweet potato, corn, cassava. Pizza and empanadas.	Drinking water. All kind of fruits. All kinds of vegetables (except starchy vegetables)

* *Empanadas consist of a thin pastry dough, either short crust or puff pastry, with a salty filling that can be meat, chicken or cold cuts with cheese, which are widely consumed in Argentina. Pastries: all with or without filling: croissants, croissants, churros, etc. Cakes and puddings: all cakes, puddings, sponge cakes, muffins, etc., homemade or commercial. SSB: sugar-sweetened beverages and ASB: artificially sweetened beverages*.

Factor scores for each pattern were then calculated and median adherence to each pattern across sociodemographic variables is shown in Table 4. For the Western pattern, greater adherence was observed in the AMBA region (*p* < 0.001), men (*p* < 0.001), and younger individuals (*p* < 0.001) as well as normal or overweight compared to obese individuals (*p* < 0.001). For the Rural pattern, a greater adherence was observed in the northeast region (*p* < 0.001), men (*p* < 0.001), younger individuals (*p* < 0.001), and with lower SEL and educational level (*p* < 0.001). Also, greater adherence was observed for normal or overweight compared with obese individuals (*p* < 0.001). Greater adherence to the Traditional dietary pattern was only observed in men (*p* < 0.001) and those with insufficient PA (*p* = 0.033). Sweet Prudent pattern showed greater adherence among women (*p* < 0.001), elder individuals (*p* = 0.002), middle or high SEL (*p* < 0.001), higher educational level (*p* < 0.001), and with sufficient PA (*p* < 0.001). Finally, there was greater adherence to the Local Western pattern in the northwest region (*p* < 0.001), men (*p* < 0.001), younger individuals (*p* < 0.001), low SEL (*p* < 0.001), and education level (*p* < 0.001) as well as individuals with insufficient PA (*p* = 0.015) and normal BMI (*p* < 0.001).

**Table 4:** Median adherence to dietary patterns according to categories of sociodemographic characteristics.

		**Western**		**Rural**		**Traditional**		**Sweet Prudent**		**Western 2**	
**Variable**	**Levels**	**Median (IQR)**	***p*-value**	**Median (IQR)**	***p*-value**	**Median (IQR)**	***p*-value**	**Median (IQR)**	***p*-value**	**median(IQR)**	***p*-value**
Sex	Male	0.18 (−0.72, 1.39)	<0.001	0.53 (−0.27, 1.33)	<0.001	0.35 (−0.42, 1.06)	<0.001	−0.22 (−0.69, 0.57)	<0.001	0.31 (−0.46, 1.11)	<0.001
	Female	−0.35 (−0.93, 0.61)		−0.31 (−0.90, 0.31)		−0.28 (−0.83, 0.47)		0.05 (−0.53, 0.69)		−0.09 (−0.69, 0.50)	
Region	AMBA	0.16 (−0.71, 1.28)^a^	<0.001	−0.07 (−0.75, 0.63)^a^	<0.001	0.05 (−0.63, 0.89)	0.622	0.03 (−0.62, 0.70)	0.074	0.11 (−0.53, 0.86)	<0.001
	Pampeana	−0.25 (−0.89, 0.96)		−0.02 (−0.70, 0.61)^a^		−0.08 (−0.76, 0.73)		−0.13 (−0.64, 0.72)		0.07 (−0.60, 0.79)	
	Cuyo	−0.24 (−0.96, 0.55)		−0.16 (−0.67, 0.40)^a^		−0.11 (−0.66, 0.62)		−0.20 (−0.58, 0.45)		−0.31 (−0.72, 0.42)	
	Northwest	−0.37 (−1.00, 0.36)^b^		0.31 (−0.45, 1.16)^b^		−0.04 (−0.75, 0.90)		−0.15 (−0.68, 0.36)		0.17 (−0.51, 0.87)	
	Northeast	−0.40 (−1.00, 0.79)^b^		0.38 (−0.67, 1.56)^b^		0.01 (−0.59, 0.74)		0.03 (−0.46, 0.73)		−0.35 (−0.96, 0.59)	
	South	−0.45 (−0.85, 0.88)		0.08 (−0.55, 1.17)^a, b^		−0.24 (−0.98, 1.02)		−0.24 (−1.04, 0.38)		−0.06 (−0.78, 0.82)	
Body mass index	<25 kg/m^2^	0.08 (−0.71, 1.14)^a^	<0.001	0.08 (−0.59, 0.96)^a^	<0.001	−0.01 (−0.66, 0.79)	0.768	−0.02 (−0.57, 0.70)	0.439	0.23 (−0.55, 0.92)^a^	<0.001
	≥ 25 to <30 kg/m^2^	−0.12 (−0.81, 0.90)^a^		0.02 (−0.72, 0.69)^a^		−0.04 (−0.73, 0.87)		−0.10 (−0.65, 0.73)		−0.05 (−0.65, 0.67)^b^	
	≥ 30 kg/m^2^	−0.43 (−1.04, 0.77)^b^		−0.16 (−0.82, 0.51)^b^		−0.08 (−0.72, 0.78)		−0.13 (−0.61, 0.56)		−0.08 (−0.70, 0.69)^b^	
Age	15–19	0.12 (−0.64, 1.22)^a^	<0.001	0.50 (−0.22, 1.14)^a^	<0.001	0.06 (−0.78, 0.82)	0.081	−0.04 (−0.47, 0.99)^a^	0.002	0.53 (−0.11, 1.23)^a^	<0.001
	20–34	0.00 (−0.78, 0.96)^a^		0.09 (−0.54, 0.97)^b^		−0.16 (−0.73, 0.87)		−0.13 (−0.67, 0.58)^b^		0.19 (−0.56, 0.93)^b^	
	35–49	−0.12 (−0.90, 1.12)^a^		−0.11 (−0.74, 0.59)^c^		0.09 (−0.55, 0.89)		−0.14 (−0.67, 0.55)^b^		0.01 (−0.60, 0.75)^b^	
	50–65	−0.48 (−1.06, 0.51)^b^		−0.28 (−0.94, 0.49)^c^		−0.12 (−0.74, 0.61)		0.07 (−0.55, 0.71)^a, b^		−0.35 (−0.89, 0.31)^c^	
Education	Primary	−0.05 (−0.87, 0.96)	0.249	0.09 (−0.58, 0.85)^a^	<0.001	−0.02 (−0.67, 0.87)	0.382	−0.21 (−0.68, 0.54)^a^	<0.001	0.11 (−0.52, 0.87)^a^	<0.001
	Secondary	−0.33 (−0.90, 0.84)		−0.30 (−0.90, 0.53)^b^		−0.10 (−0.76, 0.68)		0.24 (−0.35, 1.00)^b^		−0.34 (−0.90, 0.48)^b^	
	University	−0.14 (−0.78, 1.16)		−0.34 (−0.89, 0.37)^b^		0.04 (−0.65, 0.81)		0.17 (−0.21, 1.19)^b^		−0.33 (−1.19, 0.42)^b^	
Socioeconomic level	High	0.11 (−0.71, 1.19)	0.571	−0.11 (−0.70, 0.42)^a, b^	<0.001	−0.05 (−0.79, 0.56)	0.533	0.09 (−0.48, 1.01)^a^	<0.001	−0.09 (−0.70, 0.73)^a, b^	<0.001
	Medium	−0.11 (−0.86, 0.87)		−0.18 (−0.85, 0.66)^a^		−0.02 (−0.67, 0.78)		0.11 (−0.49, 0.84)^b^		−0.05 (−0.76, 0.69)^a^	
	Low	−0.18 (−0.89, 0.96)		0.17 (−0.54, 0.86)^b^		−0.05 (−0.70, 0.90)		−0.28 (−0.71, 0.42)^b^		0.14 (−0.48, 0.87)^b^	
Meeting physical	No	−0.07 (−0.86, 0.93)	0.580	0.05 (−0.59, 0.77)	0.205	0.06 (−0.66, 0.88)	0.033	−0.22 (−0.68, 0.51)	<0.001	0.10 (−0.56, 0.88)	0.015
activity guidelines	Yes	−0.19 (−0.88, 0.99)		−0.01 (−0.77, 0.72)		−0.13 (−0.72, 0.74)		0.07 (−0.51, 0.76)		−0.02 (−0.65, 0.72)	

a, b, c *Median values within patterns without common letters differ, p <0.05 tested with Kruskal-Wallis and post hoc p-values adjusted by BH*.

## Discussion

To the best of our knowledge, this is the first study to examine the dietary patterns of the Argentine population including all the regions of the country. The results of this exploratory FA, based on data from the ELANS study, have been able to determine five dietary patterns, which reflect the characteristics of the local diet: Western, Rural, Traditional, Sweet Prudent, and Local Western. Only Sweet Prudent dietary pattern is related to women, higher educational level, and meeting the PA recommendations. Both the Western and Rural dietary patterns are related to younger age and Local Western is associated with lower educational level. Concerning the region representation of each dietary pattern, it is worth highlighting that the Buenos Aires city and its metropolitan area grouped in the AMBA region is positively related to almost all the dietary patterns, while the region of Cuyo is negatively related to all of them. The Western dietary pattern was mainly characterized by more frequent intakes of processed meat, animal fats, refined fatty and sweet grains (pastries), SSB, pizza and empanadas, and sweets. The Rural dietary pattern includes processed meat, alcohol, SSB, bread, and other cereals and is negatively associated with ASB and fruits. The Local Western dietary pattern is characterized by pizza and empanadas, SSB, starchy vegetables, poultry, and cookies and is negatively associated with fruits, vegetables, and water intake.

The Argentinian typical food style is frequently described as monotonous and mainly based on meat, wine, potato, or other starchy vegetables and bread and other refined grains as the main foods, with lower quantities of whole grains, green vegetables, and fruits (37–39). The pattern that is named as Traditional represents this typical and local diet and it is integrated with red meat, poultry, starchy vegetables, oils, and alcohol. It is negatively associated with cereals and crackers.

The Sweet Prudent dietary pattern was characterized by more frequent intakes of vegetables, fruits and fruit juice, milk and yogurt, ASB, and sweets and cookies. It was negatively associated with infusions with sugar and baked goods. It is interesting to note that, after this FA, four of five dietary patterns correlated with SSB in this population. It has been already described and previously published that this Argentinian population consumes 15.9% of the total energy (TE) in the form of added sugars (40). The main food sources were liquids, both industrial and added to infusions (26.9 and 23.8%, respectively). The third place, with 15.4%, was represented by baked goods (bread, cookies, etc.) (41). This seems to be a pattern quite specific to Latin America and particularly to Argentina, as European countries report lower consumption of added sugars (11 to 17% of TE) and the main sources were mostly sweet products, followed by beverages (12 to 31% in adults and 20 to 34% in children), then by dairy products (42, 43).

The WHO initiatives on sugar reduction and the global awareness on sugar consumption led to replace and reformulate foods toward noncaloric sweeteners (44). A recent proposal emerged from the Ibero-American consensus on low- and no-calorie sweeteners proposed that foods and beverages with Low and no calorie sweeteners (LNCS) could be included in dietary guidelines as alternative options to products sweetened with free sugars.

Based on these environmental and educational efforts, it seems reasonable that the population represented in the healthiest (Sweet Prudent) pattern showed to consume ASB (45). It is a great concern for the Argentine population, which leads the region in added sugar consumption, to favor changes in eating habits tending to reduce the sugar content of foods and beverages (43).

Diet is one of the most studied factors and, at the same time, with the greatest potential for scientific development due to its impact on health. The quality of the diet has been associated with the pathogenesis of multiple diseases such as cardiometabolic disease, obesity, diabetes, depression, and cancer, among others (46–48).

Cena and Calder have recently described in a published review that healthy diets, in comparison with a Western diet, are higher in fruits and vegetables, whole grains, legumes, seeds, and nuts and lower in animal-based foods, particularly fatty and processed meats (49). Also, the authors highlighted that this occurs naturally in some regions of the world (i.e., Mediterranean, Dash, and Nordic diets as examples). On the contrary, the four main dietary patterns of the Argentine population—Western, Rural Traditional, and Local Western—are, as a group, based on SSB, animal fats, red and processed meats, pizza, empanadas, and refined grains with fat (baked goods and pastries).

Our findings are supported by previous studies in Argentina. A study based on intake data from 3,000 adults in two different Argentinian cities found three major dietary patterns similar to those described in this study. Traditional pattern characterized by refined grains, red meat, whole-fat dairy, and mate; healthy pattern characterized by vegetables, fruit, low-fat dairy, whole grains, and legumes; and processed-food pattern characterized by processed meat, snacks, pizza, and empanadas. Similar to our results, women and higher education levels were positively associated with healthy dietary pattern, while men younger subjects were associated with processed food (50). In a further study of a subsample of 1,983 subjects, three similar dietary patterns were identified such as Traditional, Prudent, and Convenience processed. The authors also found that markers of endothelial dysfunction were directly associated with higher adherence to Convenience-Processed dietary pattern and inversely with Prudent (51). Another study, conducted in the city of Cordoba, identified similar dietary patterns, three of them, based on meat, starchy vegetables, bakery products, oil, and mayonnaise. The main finding of this case–control study was the positive association of the Traditional, Rural, and Starchy pattern with breast cancer, while the Prudent dietary pattern showed a protective effect (20). Western diet has been associated in several meta-analyses with increased risk of all-cause mortality and increased risk of colon cancer, among others. Although the authors concluded that so far the evidence indicates that no food should be omitted from the diet in cancer patients, they highlighted the association between Western diet and increased risk of mortality (52, 53).

Western diet was also associated with obesity and nonchronic diseases including all the comorbidities derived from the increase of total body fat (54, 55). The opposite of expected, in this study, Western, Rural, and Local Western patterns, were associated with normal weight rather than obesity. On the contrary, Pou and collaborators identified in Cordoba, Argentina that the Western pattern was positively related to obesity (20). The finding of this study can be explained by the natural reason that people living with obesity (BMI ≥ 30 kg/m^2^) tend to achieve healthier diets and/or report not consuming unhealthy food and beverages (underreporting) (56, 57). In this study, we have been able to observe that the youngest adheres more to the unhealthy patterns (Western, Local Western, and Rural), while those in the older ages (50–65 years), the youngest adheres less to these patterns and more to Sweet Prudent. In agreement with the data from this study in the Spanish population, it can be observed that the older population shows a tendency toward a healthier diet, including a lower content of simple sugars and a higher content of fruits, vegetables, and legumes in comparison with the younger population (58). This is a common pattern previously described by several authors showing that younger adults tend to have less healthy diets in comparison with adults and seniors and was also previously reported in the same population (59, 60). At the same time, significant differences are observed between men and women for all the dietary patterns described, with men showing higher adherence with the Western, Local Western, Traditional, and Rural patterns, while women are positively associated with the Sweet Prudent pattern. Other authors have previously reported that the western diet (WD) style is more frequent in men than in women (61–64).

As previously described, the Argentine population has a traditional dietary history based on meats, refined flours, starchy vegetables, and SSBs. Although Argentina is an agro-producing country, vast, and with a diverse climate (65), it can be observed that the traditional dietary pattern is not particularly associated with any region and, therefore, shows a homogeneous distribution throughout the country, while the population of the Buenos Aires Metropolitan Area adheres more to the Western, Local Western, and Rural patterns.

The strength of this study is based on the precision of the data collection by two face-to-face interviews, the multiple pass methodology, and the FA applied. There are several methods to derive dietary patterns, which need to be selected according to the study questions. Dietary patterns extracted by FA do not necessarily represent healthy diets; rather, it is better suited to explore dietary patterns that exist in a given population. These patterns represent different traits in food intake and could be more interpretable than single foods or nutrients (10).

Within the limitations that we can count in this study, first, some foods, such as whole grains, fish, nuts, and seeds, are consumed by very few people in this study, which is a limitation when performing the FA to be included in any dietary pattern. Second, the analysis has been performed based on two 24-h records rather than a food frequency questionnaire and some foods of lower frequency of consumption could be less represented. As a limitation of the methodology applied, FA as well as with other posteriori methods requires subjective decisions such as grouping the foods, selecting the number of factors to retain, and their labeling. Another disadvantage of these techniques is that they generate patterns based on variation in diet, but there is no guarantee that these patterns will be predictive for a particular health outcome. The total explained variance was fairly low, only accounting for 19% of the total variance of food intake. Hence, our results should be confirmed in bigger datasets, which account for greater between and within individual variation in food intake, to improve correlation between observed variables. It could also be a limitation that this study was carried out only in urban people of big cities and limited to people under 65 years old. Taking into account the longer life expectancy, elderly population could still follow a more traditional local diet and, probably, less influenced by globalization.

Current results are reflecting the different dietary patterns of the Argentine population that, under our knowledge, have not been previously reported with this methodology. According to our results, food consumption in Argentina is expressed in a diversity of dietary patterns. Men, younger, and sedentary individuals, with lower socioeconomic and educational level, as well as individuals from the AMBA and northern regions, seem to have higher adherence to least healthy dietary patterns. We believe that in the future, it is important to consider in developing countries such as Argentina, the importance of SEL when analyzing food/ intake patterns, as it seems to be one of the main determinants. Also, although this type of methods have some limitations, in this case, the analysis reveals that Argentina can lead its food policies to favor the consumption of fruits and vegetables, decrease the consumption of sugar-containing beverages and red meat, and favor a more diverse diet. This information could be useful for policymakers to target interventions that promote better diets. Further study should confirm our findings and explore possible associations between dietary patterns and health outcomes.

## Data Availability Statement

The data analyzed in this study is subject to the following licenses/restrictions: due to ethical and legal restrictions of the institutions involved, the data underlying this study are available upon request and must be approved by the Publishing Committee of ELANS. Data are available from the ICCAS Institutional Data Access for researchers who meet the criteria for access to confidential data and also approved by the Publishing Committee of ELANS. To apply for access to these data, interested researchers must submit a detailed project proposal to the above Institution. The authors confirm that the data underlying this study will be shared provided that requests are submitted through appropriate channels. Requests for the data can be made to the Executive Secretary of the Institution. Requests to access these datasets should be directed to Mrs. Patricia Torres, ptorres@iccas.org.ar.

## Ethics Statement

The studies involving human participants were reviewed and approved by Western Institutional Review Board (# 20140605) and registered with Clinical Trials (# NCT02226627). In Argentina, the approval of the Ethics Committee of the Argentine Medical Association has been added. Written informed consent to participate in this study was provided by the participants' legal guardian/next of kin.

## Author Contributions

IK conceived and designed the study. IK and VG performed the local implementation of the study. IM and IK contributed with conceptualization. IM and MF contributed with methodology. IM, LZ, and IK wrote the original draft. MF contributed to the statistical analysis. All authors read the manuscript, revising it critically for important intellectual content, and approved the submitted version of this manuscript. The authors also state that the content has not been published elsewhere.

## Funding

The ELANS in Argentina was supported by a scientific grant from the Asociación de Fabricantes Argentinos de Coca Cola (AFAC) and support from the International Life Science Institute of Argentina and Universidad (ISALUD). The funding sponsors had no role in the design of the study, in the collection, analyses, or interpretation of data, in the writing of the manuscript, and in the decision to publish the results.

## Conflict of Interest

IK has received consulting fees from Novo Nordisk. The remaining authors declare that the research was conducted in the absence of any commercial or financial relationships that could be construed as a potential conflict of interest.

## Publisher's Note

All claims expressed in this article are solely those of the authors and do not necessarily represent those of their affiliated organizations, or those of the publisher, the editors and the reviewers. Any product that may be evaluated in this article, or claim that may be made by its manufacturer, is not guaranteed or endorsed by the publisher.
